# Association Between Single-Nucleotide Polymorphism rs2287886 of *CD209* Gene and Clinical Severity of COVID-19 in Unvaccinated Brazilian Patients

**DOI:** 10.3390/genes16091029

**Published:** 2025-08-29

**Authors:** Steffany Larissa Galdino Galisa, Raldney Ricardo Costa da Silva, Mell Cunha Aguiar, Marcus Villander Barros de Oliveira Sá, João Vinícius de Oliveira Melo, Giúlia Vitória Neves Pereira, José Rodolfo de Lima e Silva, Bianca de Lima Xavier Paiva, Andreza Gabriele da Silva Henrique, Rodrigo Feliciano do Carmo, Carlos Dornels Freire de Souza, Anderson da Costa Armstrong, Pablo Rafael Silveira Oliveira, Luydson Richardson Silva Vasconcelos

**Affiliations:** 1 Department of Parasitology, Oswaldo Cruz Foundation, Aggeu Magalhães Institute, Recife 50740-465, PE, Brazil; steffanyl39@gmail.com (S.L.G.G.); raldneyrcs@gmail.com (R.R.C.d.S.); marcusvillander@yahoo.com.br (M.V.B.d.O.S.); joaovinicius37646@gmail.com (J.V.d.O.M.); joserodolfo.silva@ufpe.br (J.R.d.L.e.S.); blima1544@gmail.com (B.d.L.X.P.); andreza.gabrieleiam@gmail.com (A.G.d.S.H.); 2 Department of Chemistry, Princeton University, Princeton, NJ 08544, USA; mellcaguiar@gmail.com; 3 Department of Biological Sciences, Federal University of Pernambuco, Recife 50670-901, PE, Brazil; giulianeves90@gmail.com; 4 Laboratório Avançado de Diagnóstico e Estudos em Saúde e Ambiente (LADESA), Colegiado de Medicina Federal, University of the São Francisco Valley (UNIVASF), Petrolina 56306-340, PE, Brazil; rodrigo.carmo@univasf.edu.br (R.F.d.C.);; 5 Institute of Biology, Federal University of Bahia, Salvador 40110-060, BA, Brazil; prsoliveira@ufba.br

**Keywords:** *CD209* gene, COVID-19, polymorphism, SARS-CoV-2

## Abstract

**Background:** Host genetic factors significantly influence individual susceptibility to severe COVID-19, potentially explaining the observed disparities in clinical outcomes across populations. One of the key effectors in innate immunity and antiviral defense is the CD209 gene. This study explored the potential correlation of the CD209 gene SNP rs2287886 with diverse COVID-19 patient outcomes. **Materials and Methods:** A total of 176 patients (87 in the moderate group and 89 in the severe/critical/death group) were included in the study. Genotyping of patients was performed using the qPCR methodology, through the TAQMAN system. The results were analyzed adopting a significance level of *p* < 0.05. **Results:** The GG genotype (compared to AG + AA) and the G allele (compared to the A allele) of the rs2287886 SNP were significantly associated with an increased severity of COVID-19 (*p* = 0.0005 and *p* < 0.0001, respectively). The G allele was more frequent in individuals with more severe clinical outcomes (49.43% vs. 25.28%). Furthermore, expression quantitative trait loci (eQTL) analysis indicated that the GG genotype of rs2287886 is associated with higher *CD209* gene expression. Furthermore, the observed interaction analysis suggests that the interactions between *CD209* and its associated proteins may play a role in modulating the immune response. **Conclusions:** Our findings suggest that Brazilian patients homozygous for the GG genotype of the rs2287886 polymorphism in the *CD209* gene may be at increased risk of severe COVID-19 in the Brazilian population and may act as a potential prognostic marker of disease severity.

## 1. Introduction

COVID-19, resulting from infection with the SARS-CoV-2 pathogen, has instigated a worldwide pandemic, causing a major impact on the population [1]. According to global surveillance data encompassing 84 countries, the prevalence of SARS-CoV-2-positive test results has shown a continuous upward trend recently [2]. On a global scale, by 15 September 2024, upwards of 776 million verified infections and in excess of 7 million deaths had been registered [3]. In Brazil, as of 18 October 2024, 5157 deaths from COVID-19 had been reported [4].

Even though there has been a reduction in the morbidity and mortality of COVID-19 compared to previous years, the SARS-CoV-2 pathogen remains subject to ongoing evolutionary processes and widespread dissemination through random mutations in the genome, some of which may confer advantages to the virus, avoiding the host immune response or showing resistance to antiviral interventions [5]. Thus, the continual appearance of novel SARS-CoV-2 variants of interest remains a worldwide issue [6].

Since COVID-19 exhibits a range of clinical presentations spanning from asymptomatic infections to fatal pneumonia and mortality events, it is of fundamental importance to study host elements that regulate the human immune response to the virus [7]. Host genetic differences are significant determinants of individual susceptibility to severe manifestations of COVID-19, potentially explaining the observed disparities in clinical phenotype between populations [1,7].

Research on single-nucleotide polymorphisms (SNPs) has shown associations with immune dysregulation, pneumonia, sepsis, and excessive cytokine release in worsening COVID-19 severity [8,9,10,11]. Furthermore, genome-wide association studies (GWASs) have identified several genetic loci associated with COVID-19 susceptibility and severity, such as 3p21.31 and 9q34.2 [12,13,14,15]. The 3p21.31 locus contains several genes encoding chemokine receptors potentially relevant to severe COVID-19. In the *CXCR6* gene, the risk allele of SNP rs71327024 was associated with the progression of severe COVID-19 due to lower gene expression in helper T cells [16]. A meta-analysis identified a group of nine highly correlated SNPs, including the *LZTFL1* and *SLC6A20* genes, which were associated with an increased risk (OR = 1.8) of severe COVID-19 [17]. Another study showed that the ABO locus mediated the risk of COVID-19 by influencing the modulation of *CD209*, one of the binding sites for the SARS-CoV-2 virus [18]. The SNP rs505922 in the *ABO* gene was replicated in four susceptibility phenotypes (infection and exposure) and one severity phenotype [12].

Innate and adaptive immunity are vital for eliminating and controlling viral infection. A major mediator of innate immunity and antiviral defense is the *CD209* gene, a receptor found on dendritic cells that participates in detecting oligosaccharides on various pathogens. Thus, polymorphisms in this gene may influence susceptibility or resistance to infection, and the severity in several infectious diseases, such as tuberculosis, severe acute respiratory syndrome, and dengue [19].

Therefore, the current research aimed to explore the potential relationship between the *CD209* gene SNP rs2287886 in patients with different COVID-19 outcomes, and according to current our knowledge, this study is the first to evaluate the association between the *CD209* SNP rs2287886 and COVID-19 severity in Brazilian patients.

## 2. Materials and Methods

### 2.1. Ethics Declaration

This study was approved by the Research Ethics Committee of the Aggeu Magalhães Institute (36403820.2.0000.5190). All patients who agreed to participate in this study signed the informed consent form. Clinical information was systematically documented using a form, while DNA samples were collected, stored, and processed in strict accordance with the ethical principles of the Declaration of Helsinki.

### 2.2. Population Characterization

This is a cross-sectional study with group comparison. In the present study, 176 patients from the state of Pernambuco, diagnosed with COVID-19 by positive RT-PCR between 2020 and 2021, were included. The individuals were classified into two main groups: moderate group (87 patients) and critical/severe group (individuals who presented severe, critical disease and patients who died) (89 patients). None of the participants were vaccinated, reflecting the limited vaccine availability during the study period.

The definition of severity of the patient groups followed the classification defined by the World Health Organization (WHO), in which critical illness is characterized by acute respiratory distress syndrome (ARDS), sepsis, septic shock, thrombosis, or conditions requiring life support; severe illness presents an oxygen saturation < 90%; severe pneumonia; severe respiratory distress (respiratory rate > 30/min); moderate disease; clinical signs of pneumonia (fever, cough, dyspnea, rapid breathing), including SpO_2_ ≥ 90%; and mild disease in the absence of any criteria for severe or critical COVID-19 [20] (World Health Organization, 2021).

### 2.3. Genomic DNA Extraction and Genotyping

Total DNA from blood samples was extracted using the ReliaPrep™ Blood gDNA Miniprep System kit, following the manufacturer’s recommendations. Then, the concentration and purity of DNA samples were quantified using a NanoDrop™ spectrophotometer (Thermo Scientific™, Waltham, MA, USA). Genotyping was performed using TaqMan genotyping assays through the SNP probe ID: rs2287886, C__11515683_1_, obtained from ThermoFisher^®^ Scientific (Thermo Fisher Scientific, Foster City, CA, USA). Real-time PCR and TaqMan genotyping assays were conducted using an Applied Biosystems Quant Studio 5 system (Thermo Fisher, Applied Biosystems, Waltham, MA, USA), following the standard PCR protocol provided by the manufacturer.

### 2.4. In Silico Tissue-Specific Gene Expression Levels and Protein Interaction Analysis

Expression quantitative trait locus (eQTL) analysis was conducted using the GTEx database (https://www.gtexportal.org/home/ accessed on 23 October 2024) to assess the association between rs2287886 genotypes and *CD209* gene expression, considering the basal expression levels of this SNP in a healthy population.

### 2.5. Protein Interactions Analysis

For gene interaction and co-expression, a protein–protein interaction (PPI) network was constructed using the online platform STRING (Search Tool for Retrieval of Interacting Genes/Proteins) version 11.5.

### 2.6. Data Analysis

Associations between categorical variables were assessed using the Chi-square test or Fisher’s test when appropriate, with Bonferroni correction applied for multiple comparisons. For quantitative variables, the *t*-test or Mann–Whitney U tests were used, based on the data distribution. Differences were considered significant with *p*-values < 0.05. To verify the existence of differences in genotypic and allelic distribution between groups, the Chi-square test was used, and Bonferroni correction was performed for multiple comparisons. Binary logistic regression was performed for all variables and corrected for possible biases, using 95% confidence intervals, performed in SPSS (v.22). The other analyses were performed using the GraphPad Prism v.8.0 program. Linkage disequilibrium (LD) analysis was performed in silico, used a suite of web-based applications (LDproxy) associated with Regulome DB RegulomeDB to explore variants in haplotype blocks and identify potential regulatory such SNPs in disease-associated loci (https://ldlink.nci.nih.gov/ accessed on 8 November 2024). Data were obtained from the 1000 Genomes Project (http://www.internationalgenome.org/ accessed 21 November 2024).

## 3. Results

### 3.1. Characteristics of the Study Population

We evaluated 176 participants, consisting of 87 individuals who had moderate COVID-19 and 89 patients who developed severe diseases. Table 1 shows the clinical and demographic data of the patients, comprising 106 men and 70 women. There were no significant differences between the two groups regarding sex (*p* = 0.0643) and age (*p* = 0.0562). The mild/moderate group had a mean age of 42 years, and the severe/critical group had a mean age of 44 years. Among the comorbidities, only obesity showed significant differences between the groups (*p* = 0.0135).

### 3.2. The rs2287886 Polymorphism and Susceptibility to COVID-19

The genotype frequencies of the severe group (χ^2^ = 2.93; *p* = 0.2305) and moderated group (χ^2^ = 4.65; *p* = 0.0977) are in Hardy–Weinberg equilibrium. However, the general population (χ^2^ = 11.06; *p* = 0.004) is not in Hardy–Weinberg equilibrium. This may be related to the sample size, and it is important to consider the various factors that can cause these deviations, such as mutations, natural selection, genetic drift, and gene flow. In an ethnically diverse population like Brazil, the observed imbalance in polymorphisms in the analyzed population may have a significant impact on disease behavior [21,22].

Table 2 shows that the genotype and allele frequencies of rs2287886 between the moderate and severe/critical groups were significantly different (*p* = 0.0005; *p* < 0.0001, respectively). The frequency of the AA and AG genotypes was higher in the moderate group (36.78% and 37.93%, respectively), and the GG genotype was higher in the most severe group (49.43%). Dominant and recessive models also showed significant differences between the groups, with the dominant model presenting a value of *p* = 0.0009 (OR = 3.401; CI = 1.667–6.815) and the recessive model a value of *p* = 0.0011 (OR = 2.889; CI = 1.493–1.662). Following Bonferroni correction, the overdominant model was no longer significant (*p* > 0.01).

### 3.3. Multivariate Logistic Regression Model

In addition, we performed a binary logistic regression considering the following independent variables: sex, age, comorbidities, and genotype (dominant and recessive models), and with the binary dependent variable being the clinical outcome (moderate/severe/critical) (Table 3). In the regression model, obesity and genotype remained significant predictors of the outcome.

For the SNP rs2287886, the dominant AG/GG model was associated with a significantly higher risk of severe disease compared to the AA genotype (OR: 3.433; *p* = 0.002 [1.586–7.431]). The recessive GG model was also associated with a significantly higher risk of severe disease (OR: 3.204; *p* = 0.001 [1.646–6.236]), although with a slightly lower odds ratio compared to the dominant model. Thus, the dominant and recessive models were significant predictors of severe/critical outcomes.

To assess the linkage disequilibrium of the rs2287886 polymorphism with other variants, we used the 1000 Genomes database (phase 3) with all populations (Africans, mixed-race Americans, East and South Asians, and Europeans) (https://ldlink.nci.nih.gov/ accessed on 8 November 2024). However, we were unable to identify any other polymorphisms or functional variants with LD r_2_ > 0.8 in relation to the SNP.

### 3.4. In Silico Tissue-Specific Gene Expression Levels to rs2287886 Polymorphism

**Figure 1:**
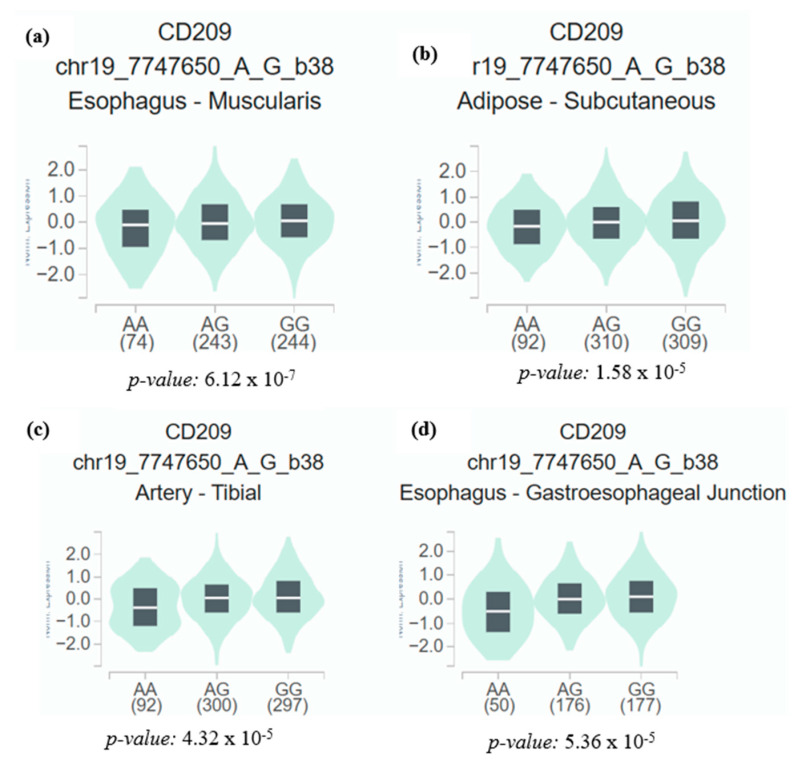
eQTL violin plot of rs2287886 variant. The genotype of rs2287886 was associated with gene expression of *CD209* gene in different tissues in GTEx database. (**a**) Esophagus, (**b**) adipose, (**c**) artery, and (**d**) esophagus.

In addition, to better understand the mechanism of susceptibility to the disease, we investigated how the rs2287886 polymorphism may affect the expression of the *CD209* gene. For this, we used the GTEx database (https://gtexportal.org/home/ accessed on 23 October 2024) for the analysis of the expression quantitative trait locus (eQTL) in different tissues. The analysis indicated that the *CD209* gene is most highly expressed in adipose, arterial, and esophageal tissues, and in all tissues, the relative expression of the gene was significantly higher in individuals with the GG genotype compared to those with the AG or AA genotype (Figure 1).

### 3.5. Protein–Protein Interaction (PPI) Network Analysis

**Figure 2:**
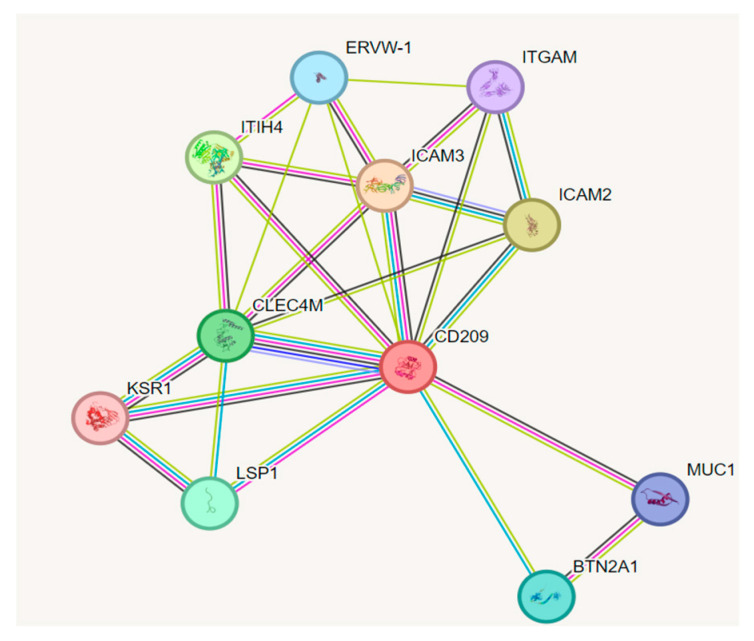
A protein–protein interaction network for the *CD209* gene showing 11 nodes. The network was visualized using STRING, with edge color intensity indicating the confidence level of functional associations.

To evaluate possible molecular associations of the CD209 protein, a PPI (protein–protein interaction) network was generated using STRING, revealing connections with 11 proteins, including CLEC4M, ICAM3, and KSR1 (Figure 2).

## 4. Discussion

This research explored the role of the rs2287886 polymorphism within the *CD209* gene in the context of severe COVID-19. Although there are some studies on the effects of rs2287886 of the *CD209* gene on some diseases, studies regarding its association with COVID-19 remain limited. In addition, there are still no data on the Brazilian population. Therefore, this study offers novel perspectives on the role of the rs2287886 polymorphism in the severe clinical presentation of COVID-19 in the Brazilian population, where data showed that the G allele of the polymorphism may be associated with a higher risk of developing severe disease.

While the immune system is crucial for combating viral infections, many individuals exhibit an exacerbated immune response [23]. The *CD209* gene contains multiple single-nucleotide polymorphisms (SNPs) that influence the expression of DC-SIGN, an important protein in immune responses [24]. In addition, *CD209* shows extensive expression in both adaptive and innate immune cells, indicating its pivotal role in immunity and possible involvement in the cytokine storm during severe SARS-CoV-2 infection [25].

Our findings suggest that the rs2287886 polymorphism in the *CD209* gene may influence COVID-19 severity. Our data show that the GG genotype was more frequent in the severity group compared to the AA genotype. Thus, the G allele at the rs2287886 locus may increase susceptibility to severe COVID-19. Furthermore, both the dominant (AG + GG) and recessive (GG) models were associated with a 3.401-fold and 3.204-fold increased risk of severe COVID-19, respectively. As shown in the multivariate analysis, obesity and *CD209* genotypes continued to influence disease severity. The association between genotypes and disease severity was stronger than that of obesity alone (dominant model *p* = 0.002; recessive model *p* = 0.001 vs. obesity *p* = 0.017). Furthermore, of the 89 patients in the severe group, only 15 were obese. Therefore, the G allele may be a significant risk factor for severe disease in most patients.

According to the study by Amraei et al., 2021, the *CD209* gene can facilitate the entry of the SARS-CoV-2 virus into the cell [26]. In addition, within the *CD209* gene promoter, sites for AP-1, Sp-1, Ets-1, and NF-kB facilitate transcriptional regulation linked to immune activation [19]. We hypothesize that the G allele of rs2287886 may correlate with increased *CD209* expression, potentially enhancing SARS-CoV-2 capture and internalization, increasing viral dissemination, amplifying inflammatory signaling, and promoting more severe disease outcomes.

The rs2287886 SNP in the *CD209* promoter region has been linked to severe dengue fever and cytomegalovirus disease, predisposition to the development of tick-borne encephalitis, and invasive pulmonary infection by Aspergillosis [27]. Furthermore, according to the literature [28], the rs2287886 polymorphism is considered a genetic factor influencing COVID-19 susceptibility in Iranians. However, the AA genotype was associated with risk, and this difference regarding our results could be explained by population-specific genetic architectures; Brazil has a strong admixture of European, African, and Amerindian ancestries, while Iranian ancestry may differ [28,29,30].

In silico analysis of *CD209* expression identified regulatory differences related to the rs2287886 polymorphism, suggesting potential implications for SARS-CoV-2 susceptibility and immune response. GTEx eQTL analysis indicated higher expression of the *CD209* gene in individuals with the GG genotype, suggesting that individuals carrying the G allele might have greater susceptibility to COVID-19 severity. High CD209 expression in the small intestine and adipose tissue, as shown by GTEx analysis, indicates potential primary sites for SARS-CoV-2 infection, and increased expression in lymphoid tissues may underlie lymphocytopenia in COVID-19 patients [31].

According to the literature, polymorphisms in gene promoter regions can alter the binding of transcription factors and the structure of cis-regulatory elements, resulting in changes in gene expression levels. These effects vary between cell types and tissues. The same variant can lead to distinct expression patterns in cells of the immune system, liver, or other tissues [32]. The pVNTR polymorphism in the promoter region of the *CYP2C9* gene was found to modulate gene expression by altering the affinity of transcription factors. These changes can impact the amount of mRNA production, influencing protein coding levels [33]. Likewise, the polymorphism (-1639G > 9) of the *VKORC1* gene, affecting the gene’s promoter activity, compromises protein production, implying a lower need for warfarin doses in individuals with the A allele, thus modulating the therapeutic response [34].

Protein–protein interaction (PPI) network analysis revealed that CD209 interacts with CLEC4M, ICAM3, and KSR1. CD209 and CLEC4M are closely related in sequence and function, thus indicating a coordinated role in the immune response [35] (The Human Protein Atlas). Lectin receptors such as CD209 and CLEC4M are frequently expressed on immune and endothelial cells and serve as pattern recognition receptors involved in the internalization and transmission of viruses such as SARS-CoV-2 [36]. Furthermore, CD209, like CLEC4M, efficiently binds to inter-cellular adhesion molecule 3 (ICAM3) and enhances T-cell infection [37]. DC-T cell interactions are essential for primary immune activation, suggesting that alterations in gene expression may impair immune function. The *KSR1* gene is a protein that also contributes to immune function in several ways, including T-cell responses, immune homeostasis, and lung defense, processes that are critical in determining the severity of COVID-19 [38]. Thus, the data suggest that the interaction between CD209 and its associated proteins may play a role in modulating immune responses, particularly T-cell activation and host defense, influencing the susceptibility and severity of COVID-19.

Identifying genetic variants associated with severe outcomes will help elucidate the processes controlling immunity, while also anticipating severe outcomes in vulnerable populations and informing therapy allocation [39]. Considering that *CD209* may act as a receptor for SARS-CoV-2, these results could enhance our understanding of genetic susceptibility to severe COVID-19.

This study has some limitations, the main one being the relatively small sample size (N = 176), which may influence the results of the observed associations. Although some genotype distributions deviate from the Hardy–Weinberg equilibrium, additional alternatives were included to strengthen the results, such as controlling for relevant covariates and functional evidence of the SNP through eQTLs and protein–protein interaction networks. Further studies with a larger number of patients are recommended to validate the findings and reduce potential biases.

Our results suggest that the GG genotype of the rs2287886 *CD209* polymorphism is associated with an increased risk of severe COVID-19 in the Brazilian population and serves as a potential biomarker. In addition, the identification of genetic factors associated with severe disease is critical for a better understanding of viral pathogenesis and clinical management.

## Figures and Tables

**Table 1:** Demographic and clinical characteristics of the 176 patients diagnosed with COVID-19.

Variables	Moderate (N = 87)	Severe (N = 89)	*p*-Value
Gender			
Male	46 (52.87%)	60 (67.41%)	0.0643
Female	41 (47.12%)	29 (32.58%)	
Age			
Mean ± SD	42.90 ± 13.34	44.77 ± 9.38	0.0562
Comorbidities			
Hypertension	15 (17.24%)	22 (24.71%)	0.2683
Diabetes	8 (9.19%)	12 (13.48%)	0.4776
Obesity	4 (4.59%)	15 (16.85%)	0.0135
Asthma	3 (3.44%)	5 (5.61%)	0.7203

**Table 2:** Frequency of alleles, genotypes, and genetic models of the rs2287886 polymorphism in the *CD209* gene in moderate and severe COVID-19 patients.

Alleles	Moderate (N = 87)	Severe (N = 89)	*p*-Value *	*p*-Value **	OR (95% IC)
A	97 (55.74%)	58 (32.58%)	<0.0001		2.606 (1.69–3.98)
G	77 (44.25%)	120 (67.41%)			
Genotype					
AA	32 (36.78%)	13 (14.60%)	0.0005	0.0025	*
AG	33 (37.93%)	32 (35.95%)			2.387 (1.06–5.47)
GG	22 (25.28%)	44 (49.43%)			4.923 (2.15–10.74)
Dominant					
AG/GG	55 (63.21%)	76 (85.39%)	0.0009	0.0036	3.401 (1.66–6.81)
AA	32 (36.78%)	13 (14.60%)			-
Recessive					
GG	22 (25.28%)	44 (49.43%)	0.0011	0.0044	2.889 (1.49–5.31)
AA/AG	65 (74.71%)	45 (50.56%)			
Overdominant					
AG	33 (37.93%)	32 (35.95%)	0.8761	3.50	0.9187 (0.509–1.66)
AA/GG	54 (62.06%)	57 (64.04%)			

* Fisher test or Chi-square when appropriate; ** Bonferroni test.

**Table 3:** Logistic regression with possible bias in dominant and recessive model to risk of severity in COVID-19.

Variables	*p*-Value	OR	CI 95%
Sex	0.131	0.606	0.316–1.16
Age	0.329	1.015	0.98–1.04
Hypertension	0.700	0.840	0.34–2.04
Diabetes	0.883	1.085	0.36–3.19
Obesity	0.017	4.870	1.33–17.81
Asthma	0.957	1.043	0.22–4.88
Dominant model: AG + GG vs. AA	0.002	3.433	1.58–7.43
Recessive model: GG vs. AA + AG	0.001	3.204	1.646–6.236

Note: Age, sex, comorbidities, and genotype are independent variables, and group (mild/moderate or severe/critical) was the dependent variable.

## Data Availability

The original contributions presented in this study are included in the article. Further inquiries can be directed to the corresponding author.
